# Biomarkers in metastatic castration-resistant prostate cancer for efficiency of immune checkpoint inhibitors

**DOI:** 10.1080/07853890.2024.2426755

**Published:** 2025-02-03

**Authors:** Zixi Wu, Junbiao Zhang, Le Li, Zhihua Wang, Chunguang Yang

**Affiliations:** a Department of Urology, Tongji Hospital Affiliated Tongji Medical College of Huazhong University of Science and Technology (HUST), Wuhan, China; b Huangshi Hubei Medical Group of Maternal and Child Health Hospital, Hubei, China

**Keywords:** Castration-resistant prostate cancer, immune checkpoint inhibitor, biomarker, PD-L1, tumour mutational burden, microsatellite instability, CDK12

## Abstract

Almost all patients with prostate cancer progress to metastatic castration-resistant prostate cancer (mCRPC) despite initial responses. In cases where traditional first-line treatments prove ineffective, the potential of immune checkpoint blockade (ICB) therapy emerges as a promising approach for managing mCRPC. However, while immune checkpoint inhibitor monotherapy or combination therapy targeting cytotoxic T lymphocyte antigen 4 (CTLA-4) and/or programmed cell death-1 (PD-1)/PD-1 ligand 1 (PD-L1) axis has been regarded as the standard therapy in many solid tumours, mCRPC as ‘cold’ tumours are considered to be relatively resistant to ICB treatment. Encouragingly, recent evidence suggests that ICB therapy may be particularly beneficial in specific subgroups of patients with high PD-L1 tumour expression, high tumour mutational burden or high tumour microsatellite instability/mismatch repair deficiency. Better understanding of these predictive biomarkers could screen which patients are most likely to benefit. This review article examines biomarkers for screening patients potentially effective in immune checkpoint inhibitor therapy.

Prostate cancer is one of the leading causes of death in men in Europe and the United States, and its incidence is second only to lung cancer [1]. Due to the lack of universal screening for prostate cancer in China, most prostate cancer patients are found at an advanced stage. Castration-resistant prostate cancer is a progressive disease of PC after androgen deprivation therapy. Although endocrine therapy is effective for a certain period, nearly all patients progress to metastatic castration-resistant prostate cancer (mCRPC) after a median survival time of 18–24 months. It is not only the most common cause of death in PC patients, but also one of the most difficult problems in the clinical treatment of PC [2]. With the rapid advancement of immunotherapy in cancer treatment, immune checkpoint blockade (ICB) therapy has achieved significant clinical success across various solid tumour types. However, for the general mCRPC population without predictive biomarker screening, independent phase II clinical trials ca184-043 [3] and ca184-095 [4] (NCT00861614, NCT01057810) both demonstrated that ipilimumab treatment did not prolong overall survival (OS) in mCRPC patients. Given the considerable variability in individual patient responses to ICB therapy, identifying reliable predictive biomarkers is crucial for personalized treatment. This review will encompass the fundamental principles and existing research on ICB therapy in prostate cancer, with a particular emphasis on a range of potential predictive biomarkers, including PD-L1 expression levels, microsatellite instability (MSI), DNA mismatch repair (dMMR), cyclin-dependent kinase 12 (CDK12) deficiency and tumour mutational burden (TMB), among others.

## PD-L1

**Figure 1.**
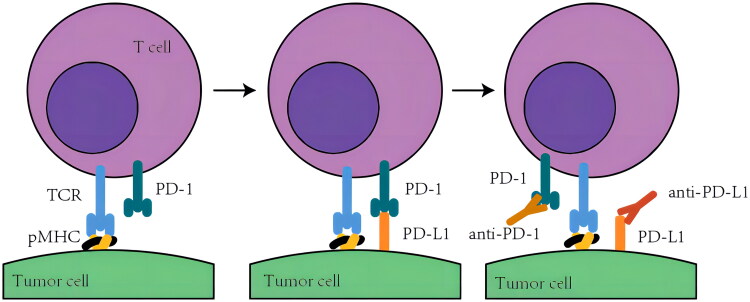
High expression of PD-L1 leads to immunosuppression and more immunotherapy targets.

In physiological conditions, the activities of T cells are intricately regulated, T cell immunity selectively eliminates pathogens and abnormal cells but avoids attacking normal cells, termed immune homeostasis [5]. Programmed cell death-1 (PD-1, which is encoded by PDCD1) and programmed cell death-ligand 1 (PD-L1, which is encoded by CD274) are vital proteins in maintaining immune homeostasis [6]. Upon binding to its ligand PD-L1 (programmed cell death 1 ligand 1), PD-1 suppresses T cell activity. This interaction governs the induction and maintenance of immune tolerance within the tumour microenvironment. However, in certain types of cancer, tumour cells can exploit this mechanism to evade immune surveillance [7]. Tumour cells may express elevated levels of PD-L1, leading to the suppression of T cell immune response during interaction with tumour cells, thereby permitting tumour growth and dissemination. At the same time, in the face of high levels of PD-L1 in the tumour microenvironment, immune checkpoint inhibitors may bring better therapeutic effects (Figure 1). Several studies have investigated the efficacy of PD-L1 expression as a predictor of treatment with immune checkpoint inhibitors (Table 1).

**Table 1.** Summary of completed studies evaluating the effect of PD-L1 as a predictor of ICIs treatment in mCRPC patients.

Trial	Treatment	Patients	Patients	End point	Results
KEYNOTE-199	Pembrolizumab	PD-L1 ≥ 1%	133	ORR OS	ORR: 5%; OS: 9.5 months
		PD-L1 < 1%	66		ORR: 3%; OS: 7.9 months
KEYNOTE-028	Pembrolizumab	PD-L1 > 1%	23	ORR	ORR: 17%
CheckMate 650	Pembrolizumab + ipilimumab	PD-L1 ≥ 1%	34	ORR PSA50	ORR: 36.4%; PSA50: 25.0%
		PD-L1 < 1%	40		ORR: 12.1%; PSA50: 7.7%
IMbassador250	Atezo + enza	PD-L1 < 1% PD-L1 ≥ 1% PD-L1 ≥ 5%	379	PFS	Longer PFS observed with PD-L1 ≥ 5%
	Enza		380		

ORR: overall response rate; OS: overall survival; PFS: progression-free survival; PSA50: PSA response rate.

The KEYNOTE-028 study [8] included 23 PD-L1-positive mCRPC patients, three-quarters of whom had previously undergone two or more treatments, revealing a 17% objective response rate (ORR), 30% disease control rate (DCR) and 37% estimated 12-month OS rate following pembrolizumab treatment, outcomes superior to those currently shown in trials employing pembrolizumab for unscreened populations.

To further investigate pembrolizumab’s antitumour activity in mCRPC patients with varying PD-L1 levels, the KEYNOTE-199 study [9] encompassed three cohorts of mCRPC patients treated with docetaxel and one or more targeted endocrine therapies. Cohorts 1 and 2 included patients with measurable PD-L1-positive and PD-L1-negative diseases, respectively, while cohort 3 enrolled patients with bone-predominant disease. All patients received pembrolizumab 200 mg, administered in 35 cycles every 3 weeks. The study included 258 patients: 133 in cohort 1, 66 in cohort 2 and 59 in cohort 3. Cohort 1 exhibited a 5% ORR (95% CI, 2–11%), while cohort 2 displayed a 3% ORR (95% CI, 1–11%). Median durations of response were not reached (1.9–21.8 months) and 10.6 months (4.4–16.8 months) for cohorts 1 and 2, respectively. DCRs were 10% in cohort 1, 9% in cohort 2 and 22% in cohort 3. Median OS was 9.5 months for cohort 1, 7.9 months for cohort 2 and 14.1 months for cohort 3. This study demonstrated that, for patients with different PD-L1 levels, pembrolizumab monotherapy did not yield differing ORRs or DCRs, but pembrolizumab provided a longer OS benefit for PD-L1-positive patients.

The combination of anti-PD-1 antibody nivolumab and ipilimumab has been studied in various malignancies, including metastatic melanoma, NSCLC and RCC. The Checkmate 650 trial [10] investigated the therapeutic effects of pembrolizumab combined with ipilimumab in 90 enrolled mCRPC patients, of whom 63 had evaluable tumour PD-L1 expression status. In patients with PD-L1 ≥ 1% vs. PD-L1 < 1%, ORR (95% CI) was 36.4% (10.9–69.2) vs. 12.1% (3.4–28.2), confirmed PSA response rate (95% CI) was 25.0% (5.5–57.2) vs. 7.7% (1.6–20.9), median rPFS (95% CI) was 5.6 (1.9–NE) vs. 3.9 (2.7–5.5) months, and OS (95% CI) was not reached (7.7–NE) vs. 15.2 (8.4–19.0) months, overall demonstrating higher treatment benefits for patients with PD-L1-positive expression when pembrolizumab and ipilimumab were combined. Concurrently, a randomized phase 3 trial, IMbassador250 [11], investigated the efficacy and safety of atezolizumab combined with enzalutamide vs. enzalutamide alone in treating mCRPC. In a sub-analysis examining PD-L1 expression, no significant differences in OS were observed for patients with PD-L1 < 1%, PD-L1 ≥ 1% or PD-L1 ≥ 5%. However, a longer PFS was associated with the use of atezolizumab and the presence of PDL1 IC2/3 expression and high levels of CD8+ T cells.

Although up to one-third of mCRPC tumours may exhibit some PD-L1 expression on tumour cells, PD-L1 expression levels are lower than in other cancers [12]. Further research is needed to determine whether PD-L1 can serve as a biomarker for the application of immune checkpoint inhibitors as monotherapy or combination therapy in mCRPC.

## Microsatellite instability-high/deficient mismatch repair

**Figure 2.**
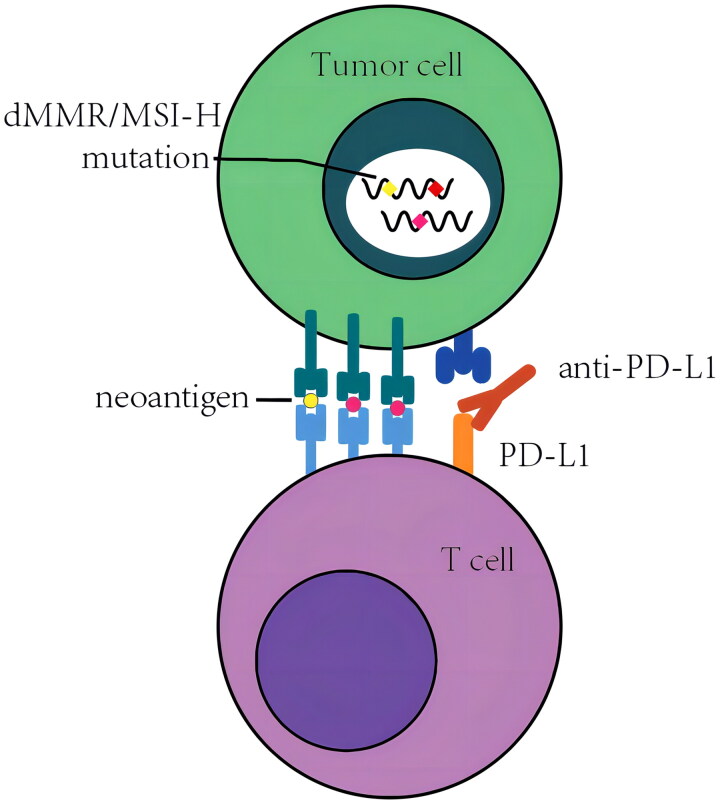
The loss of DNA mismatch repair proteins leads to the accumulation of tumour-related gene mutations and the generation of neoantigens, which triggers the host’s own anti-tumour immune response and the possibility of immunotherapy effect (CDK12, TMB-H follow a similar mechanism when used as a predictor of immunotherapy).

DNA mismatch repair is crucial to ensure the integrity of the genome. The primary function of the MMR pathway is to identify and correct base–base mismatches and insertions/deletions generated during DNA replication and recombination [13]. Characterized by pronounced MSI and defective dMMR systems, the MSI-H/dMMR molecular phenotype is common in various solid tumours [14]. Deficiencies in dMMR proteins lead to the accumulation of tumour-associated gene mutations and the generation of neoantigens, thereby eliciting the host’s anti-tumour immune response [15] (Figure 2).

In prostate cancer, the occurrence of MSI-H and dMMR is relatively rare, with a prevalence of approximately 1–3% [16] according to different studies. Multiple retrospective investigations have revealed significant outcomes for a minority of MSI-H/dMMR patients in mCRPC populations after receiving immunosuppressive therapy (Table 2).

**Table 2.** Summary of completed studies evaluating the effect of MSI-H/dMMR as a predictor of ICIs treatment in mCRPC patients.

Author	Year	Patients treated with pembrolizumab	End point	Results
Abida	2019	11	PSA50	PSA50:54.5%
Barata	2020	9	PSA50 PFS	PSA50: 44%, PFS: 9.9 months
Fang	2022	11	PSA50	PSA50: 54.5%
Sena	2021	17	PSA50 PFS	PSA50: 65%, PFS: 24 weeks
Graham	2020	15	PSA50	PSA50: 53.3%

PFS: progression-free survival; PSA50: PSA response rate.

In Abida et al.’s study [17], 11 MSI-H/dMMR castration-resistant prostate cancer patients underwent anti-PD-1/PD-L1 treatment, with six (54.5%) experiencing more than a 50% reduction in prostate-specific antigen levels and four exhibiting radiographic responses. Barata et al.’s research [18] involved nine MSI-H/dMMR castration-resistant prostate cancer patients, four of whom achieved PSA50. Meanwhile, Sena et al.’s study [19] aimed to enhance the efficacy of anti-PD1 therapy by consolidating a multicentre cohort of 65 mismatch repair-deficient (dMMR) prostate cancer patients, 19 of whom received anti-PD1 treatment. The PSA50 response rate was 65%, with a median progression-free survival (PFS) of 24 weeks (95% confidence interval 16–54). In Graham et al.’s research [20], 17 men received pembrolizumab monotherapy (anti-PD1), and of the 15 evaluable patients, eight (53%) had a PSA50 response, with seven experiencing a PSA90 decline. Fang et al. [21] and colleagues focused on the MSI-H/dMMR status in the Chinese population, analysing sequencing data from 3338 Chinese prostate cancer patients and the mutation spectrum of MMR genes. The results demonstrated that the incidence of P/LP MMR gene mutations in the cohort was higher in the American cohort (metastatic: 4.8% vs. 2.2%, *p* = .006; non-metastatic: 2.3% vs. 1.4%, *p* = .301). In this multicentre case series report, the overall efficacy of dMMR/MSI-H prostate cancer patients against PD-1 inhibitors was 54.5% (6/11).

**Figure 3.**
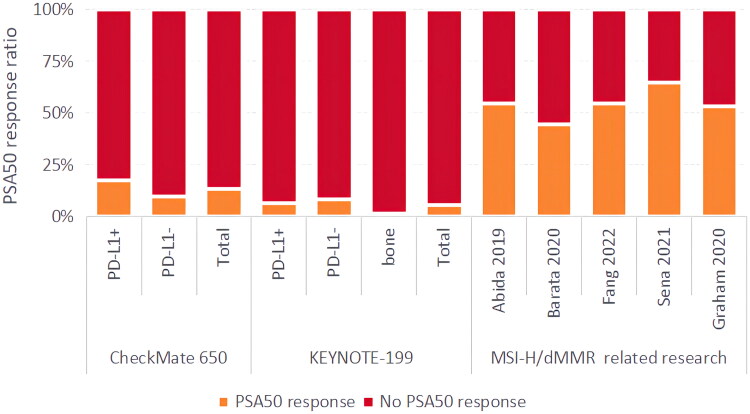
Comparison of PSA50 results between KEYNOTE-199, CheckMate 650 trials and five retrospective studies on MSI-H/dMMR, where PD-L1+ denotes PD-L1 ≥ 1%, and PD-L1− indicates PD-L1 < 1%.

In the KEYNOTE-199 and CheckMate 650 trials, patients can be regarded as representing the general population of mCRPC without prior screening for MSI-H/dMMR. In this scenario, regardless of patients’ PD-L1 status or the presence of bone metastases, it is evident that patients with MSI-H/dMMR who received ICB therapy showed better outcomes in terms of PSA50 response (Figure 3).

Pembrolizumab was approved by the FDA in 2017 for the treatment of prostate cancer with mutations in MMR and/or exhibiting MSI in tumours [22]. As we move toward personalized mCRPC treatment using multiple predictive indicators, MSI-H/dMMR will play a crucial role.

## CDK12

Cyclin-dependent kinase 12 is one of the 20 members of cyclin-dependent kinases (CDKs). Each of CDKs binds with cyclin, plays important roles in the control of cell cycle and cell division and regulates transcription in response to various cellular processes [23]. Loss-of-function mutations in CDK12 occur in 1–7% of various tumour types, with the highest prevalence in metastatic prostate cancer [24]. Preclinical data and human genomic studies suggest that CDK12 has at least two distinct roles, one associated with homologous recombination-mediated DNA repair and another related to DNA replication-coupled repair. In prostate cancer, inactivation or loss of CDK12 leads to a unique genomic instability pattern characterized by widespread focal tandem duplications throughout the genome, some of which may occur in coding regions, resulting in gene fusions and potential fusion-induced neoantigens [25].

A study [26] across nine centres identified 60 males with at least one allele (51.7% biallelic) CDK12 alterations. Nine males received PD-1 inhibitors as fourth to sixth-line systemic therapy; 33.3% of patients had a PSA response, with a median PFS of 5.4 months. Additionally, two studies reported PSA response rates to PD-1 inhibitors in patients with CDK12-deficient mCRPC. In Wu et al.’s study [27], an exceptional response (PSA decline) to anti-PD1 monotherapy was observed in two out of four mCRPC patients. In Reimers et al.’s study [28], 46 patients had CDK12 mutations; 34 had biallelic CDK12 loss (79%). In the cohort, five out of 46 CDK12 patients (10.9%) received at least one dose of anti-PD-1 checkpoint inhibitor immunotherapy, with two of them achieving a PSA50 response (Table 3).

**Table 3.** Summary of completed studies evaluating the effect of CDK12 as a predictor of ICIs treatment in mCRPC patients.

Author	Year	Patients treated with pembrolizumab	End point	Results
Antonarakis	2020	9	PSA50 PFS	PSA50: 33.3% PFS: 5.4 months
Wu	2018	4	PSA50	PSA50: 50%
Reimers	2020	5	PSA50	PSA50: 40%

PFS: progression-free survival; PSA50: PSA response rate.

Based solely on PSA50 response rates, CDK12 deficiency as a predictive marker for ICB therapy has equally exciting effects as MSI-H/dMMR. However, further research with larger sample sizes is needed to confirm its predictive efficacy in ICB therapy for mCRPC.

## TMB-H

Tumour mutational burden refers to the number of mutations in the tumour genome, typically expressed as the number of non-synonymous mutations per megabase (Mb). TMB-H (high TMB) refers to tumours with a higher mutational burden. A study [29] showed that in patients with TMB less than 10 mutations/megabase (mt/Mb), those receiving ICI treatment had a worse time to next treatment (TTNT) compared to those receiving taxane therapy. In contrast, for patients with TMB ≥10 mt/Mb, the use of ICIs was associated with a more favourable TTNT and OS compared to taxanes.

TMB-H has significant implications in cancer immunotherapy. Research has found that TMB-H tumours generally respond better to immune checkpoint inhibitors (such as PD-1/PD-L1 and cytotoxic T lymphocyte antigen 4 (CTLA-4) inhibitors). This is because a high mutational burden may generate more neoantigens, which can be recognized by the immune system as ‘non-self,’ triggering an immune response. However, a study [30] showed that compared to TMB-L, TMB-H did not improve the response rates in certain cancers, nor demonstrate a correlation between neoantigen load and CD8 T cell infiltration, such as in breast and prostate cancers. The predictive value of TMB-H for ICB therapy in mCRPC patients remains controversial and requires further investigation.

## Conclusions

So far, many studies have tried to find out the possible biological predictors for the more precise and effective application of immune checkpoint inhibitors in the treatment of specific subsets of mCRPC patients. Some of the existing studies do show good effects on specific subgroups of mCRPC patients, but inevitably have the problem of too small sample size, because whether PD-L1 positive, MSI-H/TMB-H or others, are rarely present in the population, which greatly increases the difficulty of conducting relevant studies.

Nonetheless, the application of biomarkers to screen prostate cancer subgroups would be a non-negligible step in the future. To more precisely and effectively identify and treat these patients, we must continue to search for additional, more potent biomarkers and elucidate the biological and pathological mechanisms involved. In addition, we can employ a combination of individual biomarkers to accurately discern which patients may benefit from ICB treatment. In summary, despite numerous obstacles that must be overcome, the prospects for immunotherapy in prostate cancer are indeed encouraging. At present, the integration of immunotherapy with other treatments, identification of predictive biomarkers, and determination of novel immune checkpoint targets indicate that immunotherapy will be a promising strategy in mCRPC in the coming years.

## Data Availability

The data that support the findings of this study are available from the corresponding author upon reasonable request.
